# Supplementary material for: A Vaccine with Multiple Receptor-Binding Domain Subunit Mutations Induces Broad-Spectrum Immune Response against SARS-CoV-2 Variants of Concern
Source: Vaccines (Basel). 2022 Oct 1;10(10):1653. doi: 10.3390/vaccines10101653 (PMC9609383; doi:10.3390/vaccines10101653)
Supplement: Supplementary file 1 [file vaccines-10-01653-s001.zip › vaccines-1905298-supplementary.pdf]

vaccines

MDPI

SUPPLEMENTARY MATERIALS

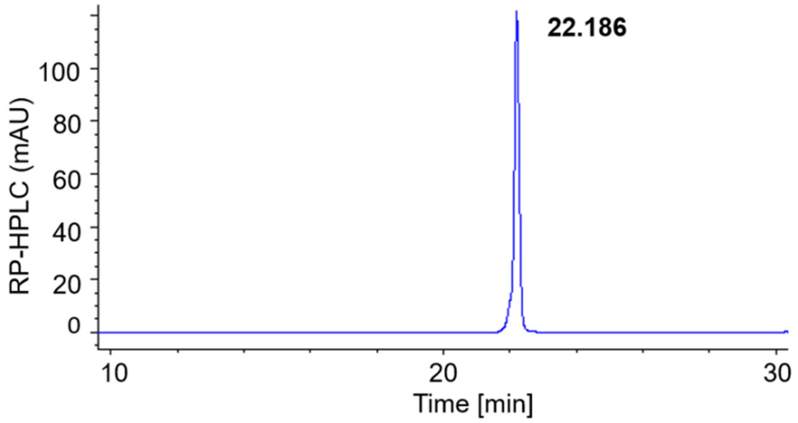

Figure S1. Purity analysis of the purified RBD5m by RP-HPLC.

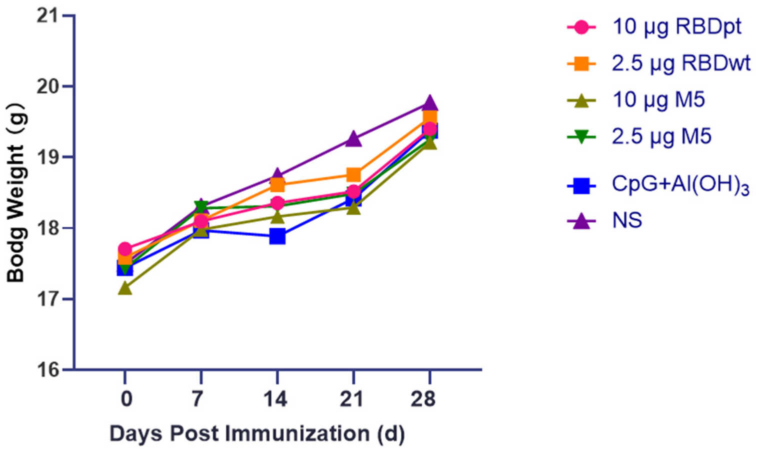

Figure S2. Body weight change of mice as a function of days post immunization. The weight of mice was monitored every 7 days after immunization. (n=10).

Table S1. Affinity constant of the RBDpt and the RBD5m.

RBD	Mean			
	$K_a(10^5Ms^{-1})$	$K_d(10^{-3}s^{-1})$	$K_D(10^{-8}M)$	$x^2(RU^2)$
**RBDpt**	4.64	5.79	1.25	0.13
**RBD5m**	4.64	5.79	1.25	0.13

K_a, association rate constant; K_d, disassociation rate constant; K_D, equilibrium disassociation constant. All of the values are determined by the ForteBio Octet™ QKe System. (n=1).
